# Dietary intake, physical activity and sedentary behaviour patterns in a sample with established psychosis and associations with mental health symptomatology

**DOI:** 10.1017/S0033291721003147

**Published:** 2023-03

**Authors:** Rebecca Martland, Scott Teasdale, Robin M. Murray, Poonam Gardner-Sood, Shubulade Smith, Khalida Ismail, Zerrin Atakan, Kathryn Greenwood, Brendon Stubbs, Fiona Gaughran

**Affiliations:** 1 Institute of Psychiatry, Psychology & Neuroscience (IoPPN), King's College London, London, UK; 2 School of Psychiatry, University of New South Wales Sydney, High St, Kensington 2033, Australia; 3 South London and Maudsley NHS Foundation Trust, Denmark Hill, London SE5 8AZ, UK; 4 Sussex Partnership NHS Foundation Trust and School of Psychology, University of Sussex, Brighton, UK

**Keywords:** Depression, diet, global functioning, negative symptoms, physical activity, psychosis

## Abstract

**Background:**

People with psychosis experience cardiometabolic comorbidities, including metabolic syndrome, coronary heart disease and diabetes. These physical comorbidities have been linked to diet, inactivity and the effects of the illness itself, including disorganisation, impairments in global function and amotivation associated with negative symptoms of schizophrenia or co-morbid depression.

**Methods:**

We aimed to describe the dietary intake, physical activity (PA) and sedentary behaviour patterns of a sample of patients with established psychosis participating in the Improving Physical Health and Reducing Substance Use in Severe Mental Illness (IMPaCT) randomised controlled trial, and to explore the relationship between these lifestyle factors and mental health symptomatology.

**Results:**

A majority of participants had poor dietary quality, low in fruit and vegetables and high in discretionary foods. Only 29.3% completed ⩾150 min of moderate and/or vigorous activity per week and 72.2% spent ⩾6 h per day sitting. Cross-sectional associations between negative symptoms, global function, and PA and sedentary behaviour were observed. Additionally, those with more negative symptoms receiving IMPaCT therapy had fewer positive changes in PA from baseline to 12-month follow-up than those with fewer negative symptoms at baseline.

**Conclusion:**

These results highlight the need for the development of multidisciplinary lifestyle and exercise interventions to target eating habits, PA and sedentary behaviour, and the need for further research on how to adapt lifestyle interventions to baseline mental status. Negative symptoms in particular may reduce patient's responses to lifestyle interventions.

## Introduction

The rates of cardiovascular disease (CVD), type 2 diabetes mellitus (T2DM) and metabolic syndrome are two-fold higher in people with psychotic disorders compared to healthy controls (Module et al., 2011; Vancampfort et al., 2015, 2016), contributing to a 10–20 year reduced life expectancy (Jayatilleke et al., 2017; John et al., 2018). This physical health disparity has been linked to side effects of antipsychotic medication, including weight gain and excessive hunger, wider determinants of health (e.g. poverty, smoking, inactivity and diet) and to the illness itself, including disorganisation, impairments in global function and amotivation associated with the negative symptoms of schizophrenia or co-morbid depression (De Hert et al., 2011; Firth et al., 2019).

A poor-quality diet, characterised by a low intake of fruit and vegetables, wholegrains, fibre and fish, and high in sodium, sugar and ultra-processed foods is associated with an increased incidence of cardiometabolic disease (Mullee et al., 2019; Srour et al., 2019), and an increased incidence of disability-adjusted life-years (DALYs) (Afshin et al., 2019; Rico-Campà et al., 2019). In 2017, 255 million DALYs were attributed to poor dietary intake globally, with the leading dietary risk factors being low intake of whole grains (82 million DALYs), high sodium intake (70 million DALYs) and low fruit intake (65 million DALYs) (Afshin et al., 2019). Additionally, low levels of physical activity (PA) and high levels of sedentary behaviour are associated with increased cardiometabolic disease risk, and related premature mortality (Garber et al., 2011; Lear et al., 2017). In a sample of 130 000 people from 17 countries, meeting PA guidelines was associated with hazard ratios of 0.72 (0.67–0.77) for mortality, and 0.80 (0.74–0.86) for major CVD (Lear et al., 2017).

People with psychosis are less likely to meet the World Health Organisation International Food Consumption Recommendations (WHO-IFCR) (⩾5 portions of fruit/vegetables, 30 g fibre and <5 g salt per day; <10% of daily calorie intake in saturated fat) (WHO, 2015) than are the general population, with poorer diet quality linked to the chronicity of psychosis (Dipasquale et al., 2013; Firth et al., 2018a; Scoriels et al., 2019; Teasdale et al., 2019). Poorer diet quality may be associated with increased appetite due to antipsychotic medication, and with amotivation and disorganisation which may impede the ability to plan and prepare meals (Teasdale et al., 2019). Moreover, people with psychosis spend an average of 12.6 h per day sedentary (Stubbs, Williams, Gaughran, & Craig, 2016b; Vancampfort et al., 2017), and only 56.6% meet the World Health Organisation's (WHO) (WHO, 2010) recommendations of 150 min of PA per week (Stubbs et al., 2016a).

Despite the increased focus on physical health in people with mental illness, there is a paucity of evidence exploring the relationship between dietary intake and symptomatology or global function in people with psychosis. This includes a paucity of well-designed prospective studies, further limited by over reliance on unvalidated tools to measure dietary intake, thereby reducing the generalisability of findings (Teasdale et al., 2019). Moreover, there is minimal research exploring the intersectionality of risks between mental health and PA and sedentary behaviour, and lack of longitudinal data in those with severe mental illnesses (SMI).

The Improving Physical Health and Reducing Substance Use in Severe Mental Illness (IMPaCT) Programme was set out to determine the extent of physical health and lifestyle risks in people with psychosis and to evaluate a health promotion intervention (Gaughran et al., 2020). IMPaCT highlighted the variation in PA in people with established psychosis, with 44% engaging in low-intensity, 44% in moderate and 12% in high intensity PA, with the level of PA associated with cardiometabolic risk markers including obesity and dyslipidaemia. Similar variations were evident in relation to dietary fat and fibre intake.

In this paper we aimed to expand this by:

1. Describing in greater detail the reported dietary intake, PA and sedentary behaviour patterns over time of patients with established psychosis participating in the IMPaCT randomised controlled trial (RCT) (baseline, and 12- and 15-month follow-ups) using data generated from tools validated for use in primary care and/or schizophrenia.
2. Testing the hypothesis that there would be a relationship between negative symptoms, depression severity and global function with PA, sedentary behaviour and diet, in particular fruit and vegetable intake, at study baseline and again at 12-month follow-up (at completion of the supervised intervention).
3. Testing the hypothesis that there would be a relationship between negative symptoms, depression severity and global function at study baseline with PA, sedentary behaviour and fruit and vegetable intake 12-months later, and with change in these lifestyle factors from study baseline to 12-month follow-up.

## Methods

### Study design

This is a secondary analysis of data from the IMPaCT RCT (Gaughran et al., 2017) which took place between 2010 and 2016 and was part of a National Institute for Health Research (NIHR) funded Programme (RP-PG-0606-1049). Participants were randomised to receive a module-based, health promotion intervention (IMPaCT therapy), through their care coordinator in addition to usual mental health care delivered by care coordinators (TAU) or to TAU alone. All care coordinators were offered a 1-h training session in best practice for physical health awareness to ensure standardized TAU. IMPaCT therapy used motivational interviewing (MI) and cognitive behavioural therapy (CBT) to address lifestyle choices and support behaviour change in key areas including exercise, diet, tobacco smoking, alcohol use, cannabis use and T2DM. Care coordinators received 4 days training in delivering the modular intervention and fortnightly supervision. IMPaCT therapy showed no significant effect on the physical or mental health subscales of the short form-36 (SF-36) questionnaire *v.* TAU at 12 or 15 months. No effect was observed for cardiovascular risk indicators, except for high-density lipoprotein cholesterol, which improved more with IMPACT therapy compared to TAU, demonstrating the challenges of reducing cardiovascular risk among those with established psychosis (Gaughran et al., 2017). Full details of the study, setting, recruitment and outcome measures have been reported elsewhere (Gaughran et al., 2013, 2017, 2020). Ethical approval was obtained from the South London and Maudsley and the Institute of Psychiatry NHS Ethics Committee (REC Ref. no. 09/HO80/41). Dietary intake and behaviour patterns of the IMPaCT sample have been previously reported (Gardner-Sood et al., 2015; Gaughran et al., 2017) although longitudinal results have not been reported, and no correlational analyses between these factors and mental health symptomology, and global function, have been conducted.

### Setting

Participants were recruited from community mental health teams in five mental health NHS trusts across England (Gaughran et al., 2013, 2017).

### Subjects

The inclusion criteria were as follows: aged 18–65 years; a primary diagnosis of psychotic illness [meeting International Classification of Diseases (ICD)-10 diagnosis criteria and encompassing: F20–29, including schizophrenia, schizoaffective disorder, bipolar affective disorder and delusional disorder, F31.2, F32.3 and F33.3], and capacity to provide informed consent. The exclusion criteria included: a primary diagnosis of intellectual disability; a first-episode psychosis (FEP); a physical health condition (such as cancer), that could independently influence metabolic measures; pregnant or less than 6 months postpartum; or a life-threatening/terminal medical condition (Gaughran et al., 2013).

### Data collection

Demographic data including age, gender, ethnicity and educational attainment were extracted via patient records and interview with the participant. Mental health was measured using the Positive and Negative Syndrome Scale (PANSS; Kay, Opler, and Lindenmayer, 1989), the Montgomery–Asberg Depression Rating Scale (MADRS; Montgomery and Asberg, 1979) and the Global Assessment of Functioning (GAF; American Psychiatric Association, 2012) through face-to-face interview.

### Co-primary outcomes

The co-primary outcomes of interest in this paper were dietary intake and PA at baseline, 12-months and 15-months. Dietary intake was assessed using a modified version of the self-report Dietary Instrument for Nutrition Education (DINE; Roe, Strong, Whiteside, Neil, and Mant, 1994). The DINE has been validated for use in primary care (Roe et al., 1994) and has been used in populations with SMI without documented issues with feasibility (Attux et al., 2013; Brown & Chan, 2006; Osborn et al., 2018). The DINE is a semi-quantitative food frequency questionnaire which includes 19 food groups. A modified version of the DINE was utilised to overcome limitations relating to the original DINE including inaccuracy of absolute nutrient values and lack of details regarding specific foods. The modified version utilised in this study included all questions from the original DINE, with an additional seven questions across 17 food categories, commonly consumed by the population under investigation, including combined fresh fruit and vegetable intake, salt use, soft drinks and hot beverages (online Supplementary Appendix One). The primary dietary outcome was a combined intake of fresh fruit and vegetables; this outcome was added to the DINE questionnaire as a supplementary question and categorised into three groups (⩽3 per month, 1–4 weekly, ⩾5 weekly) for feasibility of analysis.

PA was assessed via the self-report International Physical Activity Questionnaire (IPAQ; Craig Marshall et al., 2003). The IPAQ has been validated for the assessment of activity patterns in schizophrenia (Faulkner, Cohn, & Remington, 2006). For each respondent, the number of minutes spent over the past 7 days participating in moderate and/or vigorous activity was collected and categorised into two groups (<150 min of moderate and/or vigorous activity per week; ⩾150 min of moderate and/or vigorous activity per week). Sedentary behaviour was quantified as minutes spent sitting per day.

### Statistical analysis

Data analysis was conducted using Statistical Package for the Social Sciences (SPSS) version 25 (Chicago, USA).

First, descriptive measures were presented, by treatment arm, for demographic and clinical variables as well as dietary intake, PA and sedentary behaviour at baseline and 12- and 15-month follow-ups, and the distribution of data observed. Missing data were treated as missing.

Next, we assessed the association between mental health (PANSS negative symptoms score, MADRS score and GAF score) and (1) fresh fruit and vegetable intake (⩽3 per month, 1–4 weekly, ⩾5 weekly), (2) PA (below or above the recommended 150 min of moderate and/or vigorous activity per week) and (3) minutes of sedentary behaviour per day, at baseline and 12 months. A logistic regression model was employed to assess the association between mental health and fruit and vegetable intake, and PA. A linear regression model was used to assess the association between mental health and sedentary behaviour. A regression model was performed adjusting for age, ethnicity, gender and educational attainment. For the 12-month follow-up separate analyses were conducted for the two treatment arms.

Following this, we assessed the association between baseline mental health and (1) fresh fruit and vegetable intake, (2) PA and (3) sedentary behaviour at 12 months, and changes in 1–3 from baseline to 12 months using logistic and linear regression models adjusting for confounders as above and separated by treatment arm.

To account for multiple testing an adjusted *p* value ⩽0.006 was used for significance (Bland & Altman, 1995). To obtain this value the standard *p* value of 0.05 was divided by 9 to account for analysis of three mental health measures and three lifestyle variables.

## Results

### Basic characteristics

This secondary analysis used baseline data from 406 randomly selected patients with established psychosis, with follow-up data from 318 participants and 301 participants who completed 12-month and 15-month follow-ups, respectively.

Overall, 406 participants were randomised as part of the IMPaCT RCT, 213 to the treatment arm (IMPaCT therapy) (mean age: 43.8 ± 10.1, 55% male) and 193 to TAU (mean age: 44.7 ± 10.2, 61% male). The most frequent ethnicity was Caucasian (55.1%), followed by Black African or Black Caribbean (34%). Eighty-eight percent resided in urban locations and 73.2% were educated to at least General Certificate of Secondary Education (GCSE) level (or equivalent). Mental health symptom severity was similar between groups at baseline. Additional details regarding main clinical characteristics are presented elsewhere (Gaughran et al., 2017).

#### Descriptive analysis: dietary intake, PA and sedentary behaviour

Three-hundred ninety-seven participants completed the DINE questionnaire at baseline, and 310 and 293 participants completed the DINE questionnaire at 12- and 15-month follow-ups, respectively (data were missing from several participants due to the length of the questionnaire/time required for completion). At baseline, 58% reported consuming ⩾5 servings of fresh fruit and/or vegetables a week, with 53% consuming ⩾3 servings of fruit per week and 58% consuming ⩾3 servings of vegetables per week. In reference to discretionary foods: 41% reported consuming ⩾3 servings of biscuits, chocolate or crisps per week, 31% drank ⩾500 ml of fizzy drinks per day and 18% reported consuming ⩾3 servings of fried food each week. Similar patterns in dietary intake were observed at 12- and 15-month follow-ups.

All 406 participants provided data at baseline regarding their PA in the previous week, and all 318 and 301 participants provided PA data at 12- and 15-month follow-ups, respectively. At baseline, 82% reported taking no vigorous PA and 42% reported no moderate PA. Additionally, 22% reported walking for ⩾10 min on one or less days. Only 29% reported completing ⩾150 min of moderate and/or vigorous activity per week. The average amount of time spent sitting was 495 ± 235 min per day or 8.3 h, and 72% spent ⩾6 h per day sitting. Similar patterns in PA were observed at 12- and 15-month follow-ups.

**Fig. 1.**
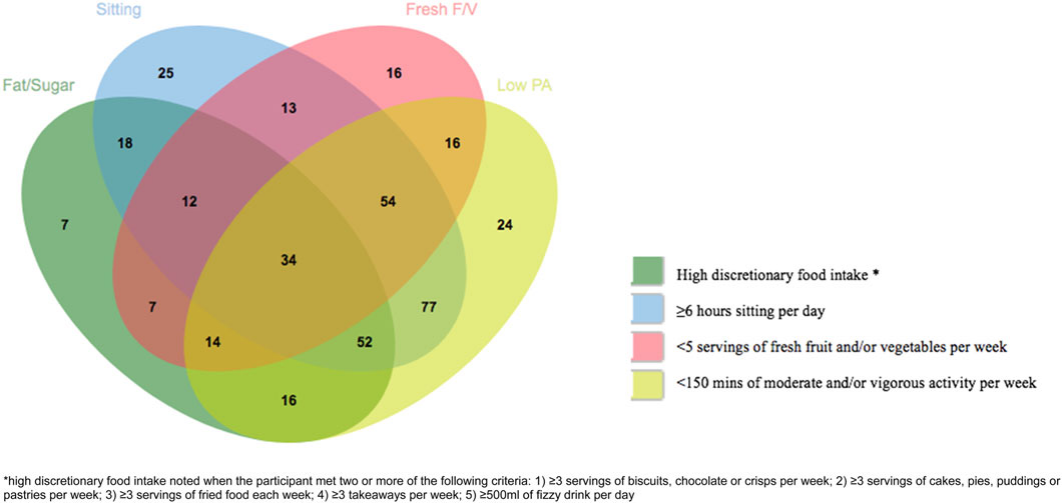
Illustration of the relationship between dietary quality, PA and sedentary behaviour.

A detailed description of dietary intake and PA at baseline and 12-month and 15-month follow-up is provided in Table 1. Figure 1 demonstrates the relationship between dietary quality, PA and sedentary behaviour at baseline.

**Table 1.** Diet and PA characteristics of the study population

Lifestyle component		Baseline			12 months			15 months		
		All	IMPaCT	TAU	All	IMPaCT	TAU	All	IMPaCT	TAU
Fresh fruit and vegetables	⩽3 servings per month, %	11.1	11.5	10.6	10.3	10.1	10.5	11.9	12.8	11.0
	1–4 servings per week, %	30.7	33.0	28.2	24.5	26.6	22.4	28.7	31.8	25.6
	⩾5 servings per week, %	58.2	55.5	61.2	65.2	63.3	67.1	59.4	55.4	63.5
Fruit^a^	⩽2 servings per week, %	46.6	50.7	42.0	39.8	44.3	35.1	48.1	52.0	44.2
	3–5 servings per week, %	15.6	16.3	14.9	22.3	22.5	23.2	17.7	14.9	20.7
	⩾6 servings per week, %	37.8	33.0	43.1	37.9	35.8	41.7	34.2	33.1	35.1
Vegetables^b^	⩽2 servings per week, %	42.1	42.1	42.0	42.5	41.1	44.0	45.1	47.3	42.8
	3–5 servings per week, %	26.4	27.8	25.0	24.4	27.2	21.3	22.9	20.9	24.8
	⩾6 servings per week, %	31.5	30.1	33.0	33.1	31.6	34.6	32.0	31.8	32.4
Fried food^c^	<1 serving per week, %	41.1	42.6	39.4	38.2	36.7	39.7	49.1	48.0	50.3
	1–2 servings per week, %	41.1	41.6	40.4	41.4	42.4	40.4	35.2	40.5	29.7
	⩾3 servings per week, %	17.9	15.8	20.2	20.4	20.9	19.9	15.7	11.5	20.0
Cakes, pies, puddings	<1 serving per week, %	39.0	41.6	36.2	44.0	44.9	43.0	44.4	46.0	42.8
	1–2 servings per week, %	37.8	36.4	39.4	30.4	32.9	27.8	31.7	32.4	31.0
	⩾3 servings per week, %	23.2	22.0	24.5	25.6	22.2	29.1	23.9	21.6	26.2
Biscuits, chocolate, crisps	<1 serving per week, %	30.0	29.7	30.3	28.2	28.5	27.8	33.1	35.9	30.3
	1–2 servings per week, %	29.0	28.2	29.8	28.8	25.3	32.5	22.9	18.2	27.6
	⩾3 servings per week, %	41.1	42.1	39.9	43.0	46.2	39.7	44.0	45.9	42.1
Fish (not fried)	<1 serving per week, %	47.6	51.2	43.6	48.9	53.2	44.4	45.2	46.9	43.4
	⩾1 serving per week, %	52.4	48.8	56.4	51.1	46.8	55.6	54.8	53.1	56.6
Fizzy drinks	<500 ml per day, %	68.8	68.4	69.1	73.2	70.3	76.3	74.6	71.6	77.8
	⩾500 ml per day, %	31.2	31.6	30.9	26.8	29.7	23.7	25.4	28.4	22.2
Takeaways	⩽3 per month, %	51.4	53.1	49.5	53.5	49.1	58.3	53.1	56.2	50.0
	1–2 per week, %	33.5	32.5	34.6	33.2	36.5	29.8	33.4	32.9	34.0
	⩾3 per week, %	15.1	14.4	16.0	13.2	14.5	11.9	13.4	11.0	16.0
Vigorous PA	0 days in the last week, %	81.5	82.6	80.3	87.1	86.8	87.4	84.1	86.2	82.0
	1–2 days in the last week, %	10.3	8.9	11.9	6.3	5.0	7.5	8.6	5.9	11.4
	⩾3 days in the last week, %	8.1	8.5	7.8	6.6	8.2	5.0	7.3	7.9	6.6
Moderate PA	0 days in the last week, %	41.9	44.1	39.4	41.6	47.5	35.8	47.0	53.3	40.7
	1–2 days in the last week, %	32.7	30.5	35.2	32.2	27.2	37.1	31.8	28.3	35.3
	⩾3 days in the last week, %	25.3	25.4	25.4	26.2	25.3	27.0	21.2	18.4	24.0
Walking for ⩾10 min or more	⩽1 day in the last week, %	21.5	22.1	20.8	25.5	27.0	23.9	24.5	23.0	26.0
	2–6 days in the last week, %	34.3	33.8	34.9	31.8	33.3	30.2	29.1	25.7	32.7
	7 days in the last week, %	44.2	44.1	44.3	42.8	39.6	45.9	46.4	51.3	41.3
Percentage meeting 150 min recommended activity per week, %		29.3	29.1	29.5	27.4	22.8	32.1	22.5	19.1	26.0
Mean time spent sitting per day (min) [s.d.]		495.4 [234.5]	511.5 [236.4]	477.8 [231.8]	486.2 [229.7]	492.1 [240.1]	480.4 [219.5]	487.1 [257.2]	501.6 [276.1]	472.5 [236.7]
Percentage sitting for ⩾6 h per day, %		72.2	76.7	67.2^d^	71.2	70.7	71.7	65.9	65.5	66.2

a Including fresh, frozen and canned fruit.

b Including fresh, frozen and canned, and excluding peas, beans and lentils.

c Including fish, chips, cooked breakfasts and samosas.

d Significant difference between IMPaCT and TAU groups [χ^2^(1, *N* = 395) = 4.430, *p* = 0.043].

#### Associations between mental health symptoms and diet, PA and sedentary behaviour (Table 2)

At baseline, those with higher PANSS negative symptom scores and greater depression severity were more likely to consume ⩽3 servings of fresh fruit and/or vegetables each month than to consume ⩾5 servings of fresh fruit and/or vegetables a week [odds ratio (OR) 1.10, 95% confidence interval (CI) 1.03–1.17; and OR 1.05, 95% CI 1.02–1.09 respectively], although this did not withstand correction for multiple testing. These associations were not observed at 12 months and no association between global function and fresh fruit and vegetable intake was found (Fig. 1).

**Table 2.** Cross-sectional associations between mental health symptoms and dietary intake, PA and sedentary behaviour at baseline and 12-month follow-up

		Base PANSS negative	Base MADRS	Base GAF
Baseline				
Fruit and vegetables		*R*^2^ = 0.095, χ^2^ = 7.080, *p* = 0.029*	*R*^2^ = 0.095, χ^2^ = 8.702, *p* = 0.013*	*R*^2^ = 0.073, χ^2^ = 1.263, *p* = 0.532
PA		*R*^2^ = 0.076, χ^2^ = 3.336, *p* = 0.068	*R*^2^ = 0.072, χ^2^ = 3.197, *p* = 0.074	*R*^2^ = 0.077, χ^2^ = 4.718, *p* = 0.030*
Sedentary behaviour		*β* = −0.015, *B* = −0.743, *p* = 0.774	*β* = 0.118, *B* = 2.939, *p* = 0.024*	***β* = −0.171, *B* = −3.010, *p* = 0.001**
		FU PANSS negative	FU MADRS	FU GAF
12 months				
Fruit and vegetables	IMPaCT	*R*^2^ = 0.092, χ^2^ = 2.173, *p* = 0.337	*R*^2^ = 0.088, χ^2^ = 1.547, *p* = 0.461	*R*^2^ = 0.078, χ^2^ = 0.254, *p* = 0.881
	TAU	*R*^2^ = 0.193, χ^2^ = 2.293, *p* = 0.242	*R*^2^ = 0.087, χ^2^ = 0.956, *p* = 0.318	*R*^2^ = 0.194, χ^2^ = 2.348, *p* = 0.309
PA	IMPaCT	***R*^2^ = 0.208, χ^2^ = 8.502, *p* = 0.004**	*R*^2^ = 0.140, χ^2^ = 0.740, *p* = 0.390	*R*^2^ = 0.135, χ^2^ = 0.285, *p* = 0.594
	TAU	*R*^2^ = 0.136, χ^2^ = 4.698, *p* = 0.030*	*R*^2^ = 0.106, χ^2^ = 0.680, *p* = 0.410	*R*^2^ = 0.139, χ^2^ = 3.554, *p* = 0.059
Sedentary behaviour	IMPaCT	*β* = 0.036, *B* = −1.859, *p* = 0.661	*β* = 0.212, *B* = 5.462, *p* = 0.009*	*β* = −0.149, *B* = −3.120, *p* = 0.072
	TAU	*β* = 0.046, *B* = 1.855, *p* = 0.576	*β* = 0.050, *B* = 1.191, *p* = 0.539	*β* = −0.199, *B* = −3.461, *p* = 0.016*

FU, 12-month follow-up; PANSS, The Positive and Negative Syndrome Scale; MADRS, Montgomery–Asberg Depression Rating Scale; GAF, global assessment of functioning; TAU, treatment as usual. Associations that meet the standard p value for significance (*p* ≤ 0.05) are given in bold.

*Does not withstand Bonferroni-adjusted *p* value of ⩽0.006.

At baseline, those with higher global function were more likely to meet the recommended PA guideline than to not meet this guideline (OR 1.02, 95% CI 1.00–1.04), although this association did not withstand correction for multiple testing. This cross-sectional association persisted in the TAU group at 12 months (OR 1.03, 95% CI 1.00–1.06) but in the IMPaCT therapy group, the relationship between global function and meeting PA guidelines was no longer apparent, this association did not withstand correction for multiple testing. At 12 months, those with greater PANSS negative symptoms were less likely to meet the recommended PA guideline than to not meet this guideline although that relationship had not been evident at baseline (baseline: OR 0.95, 95% CI 0.91–1.01; 12 months: IMPaCT therapy: OR 0.86, 95% CI 0.76–0.96, TAU: 12 months: OR 0.92, 95% CI 0.85–1.00) and this association withstood correction for multiple testing in the IMPaCT group only. No association between depression severity and PA was found.

There was a negative association between global function and sedentary behaviour at baseline (baseline: *β* = −0.17, 95% CI −4.78 to −1.24), which withstood correction for multiple testing. This cross-sectional association persisted in the TAU group at 12 months (TAU: *β* = −0.20, 95% CI −6.27 to −0.65) along with a positive association between depressive symptoms and sedentary behaviour at baseline and, in the IMPACT therapy group only, at 12 months (baseline: *β* = 0.12, 95% CI 0.39–5.49; 12 months: IMPaCT: *β* = 0.21, 95% CI 1.40–9.53), although these associations did not withstand correction for multiple testing. There was no association between negative symptoms and sedentary behaviour.

#### Associations between mental health symptoms at baseline and diet, PA and sedentary behaviour at 12 months (Table 3)

No significant associations between baseline mental health symptoms and either diet or sedentary behaviour at 12 months were observed in either treatment arm.

**Table 3.** Association between mental health symptoms at baseline and dietary intake, PA and sedentary behaviour at 12-month follow-up, and change in dietary intake, PA and sedentary behaviour from baseline to 12-month follow-up

		Base PANSS negative	Base MADRS	Base GAF
12 month fruit and vegetables	IMPaCT	*R*^2^ = 0.114, χ^2^ = 5.164, *p* = 0.076	*R*^2^ = 0.090, χ^2^ = 1.520, *p* = 0.468	*R*^2^ = 0.079, χ^2^ = 0.253, *p* = 0.881
	TAU	*R*^2^ = 0.183, χ^2^ = 0.482, *p* = 0.786	*R*^2^ = 0.185, χ^2^ = 1.021, *p* = 0.600	*R*^2^ = 0.184, χ^2^ = 0.967, *p* = 0.617
12 month PA	IMPaCT	***R*^2^ = 0.198, χ^2^ = 7.648, *p* = 0.006**	*R*^2^ = 0.140, χ^2^ = 1.130, *p* = 0.288	*R*^2^ = 0.141, χ^2^ = 1.337, *p* = 0.248
	TAU	*R*^2^ = 0.134, χ^2^ = 3.699, *p* = 0.054	*R*^2^ = 0.103, χ^2^ = 0.003, *p* = 0.959	***R*^2^ = 0.184, χ^2^ = 9.880, *p* = 0.002**
12 month sedentary behaviour	IMPaCT	*β* = −0.053, *B* = −2.566, *p* = 0.533	*β* = 0.130, *B* = 3.282, *p* = 0.114	*β* = −0.034, *B* = −0.670, *p* = 0.681
	TAU	*β* = 0.092, *B* = 4.556, *p* = 0.285	*β* = 0.061, *B* = 1.543, *p* = 0.455	*β* = −0.134, *B* = −2.174, *p* = 0.098
Change in fruit and vegetables	IMPaCT	*R*^2^ = 0.207, χ^2^ = 4.892, *p* = 0.180	*R*^2^ = 0.211, χ^2^ = 5.583, *p* = 0.134	*R*^2^ = 0.238, χ^2^ = 10.329, *p* = 0.016*
	TAU	*R*^2^ = 0.187, χ^2^ = 5.530, *p* = 0.137	*R*^2^ = 0.151, χ^2^ = 2.145, *p* = 0.543	*R*^2^ = 0.138, χ^2^ = 0.128, *p* = 0.988
Change in PA	IMPaCT	***R*^2^ = 0.300, χ^2^ = 13.153, *p* = 0.004**	*R*^2^ = 0.241, χ^2^ = 2.723, *p* = 0.436	*R*^2^ = 0.248, χ^2^ = 4.087, *p* = 0.252
	TAU	*R*^2^ = 0.177, χ^2^ = 4.115, *p* = 0.249	*R*^2^ = 0.149, χ^2^ = 4.21, *p* = 0.936	*R*^2^ = 0.218, χ^2^ = 12.076, *p* = 0.007*
Change in sedentary behaviour	IMPaCT	*β* = 0.028, *B* = 1.586, *p* = 0.751	*Β* = −0.065, *B* = −1.936, *p* = 0.443	*β* = 0.092, *B* = −2.130, *p* = 0.282
	TAU	*β* = −0.116, *B* = −6.869, *p* = 0.191	*β* = 0.140, *B* = 4.176, *p* = 0.092	*β* = −0.053, *B* = −1.037, *p* = 0.519

PANSS, The Positive and Negative Syndrome Scale; MADRS, Montgomery–Asberg Depression Rating Scale; GAF, global assessment of functioning; TAU, treatment as usual. Associations that meet the standard p value for significance (*p* ≤ 0.05) are given in bold.

*Does not withstand Bonferroni-adjusted *p* value of ⩽0.006.

In the IMPaCT group, those with greater baseline PANSS negative symptoms were more likely to not meet the recommended PA guideline at 12 months than to meet this guideline (OR 1.16, 95% CI 1.03–1.30). Moreover, in the TAU group, those with higher global function at baseline were more likely to meet than not meet the recommended PA guideline at 12 months (OR 1.05, 95% CI 1.02–1.08), while those with lower global function at baseline were less likely to meet the recommended PA guidelines at 12 months. This relationship was not found in the IMPaCT therapy group. All these associations withstood correction for multiple testing.

#### Associations between mental health symptoms at baseline and change in diet, PA and sedentary behaviour over 12 months (Table 3)

Those in the IMPaCT group with greater baseline global function were less likely to increase their fresh fruit and vegetable intake over the following year than to continue their baseline level of fresh fruit and vegetables intake, whether that be ⩾5 (OR 0.94; 95% CI 0.91–0.98) or <5 servings a week (OR 0.94; 95% CI 0.90–0.99). Those with lower global function at baseline were more likely to increase their fresh fruit and vegetable intake over the following year than to continue with their baseline level of fresh fruit and vegetables intake, but this association did not withstand correction for multiple testing. Baseline function had no effect on likely change in fresh fruit and vegetable intake in the TAU group. No significant associations between baseline depression severity or negative symptoms and subsequent change in fruit and vegetable intake were found.

Those in the IMPaCT group with higher baseline PANSS negative symptoms were more likely to remain exercising below clinical guidelines over the year than to increase their PA over 12 months from below to meeting recommendations (OR 1.29, 95% CI 1.07–1.55, *p* = 0.008). Those with lower baseline PANSS negative symptoms were more likely to increase their PA over 12 months from below to meeting recommendations than to remain exercising below guidelines over the year, and this association withstood correction for multiple testing. Baseline negative symptoms did not affect the likelihood of changing activity category in the TAU group. Those in the TAU group with higher global function at baseline were more likely to increase their activity level from below to meeting recommendations over 12 months (OR 1.05, 95% CI 1.01–1.09) or to meet clinical guidelines at both baseline and 12 months (OR 1.06; 95% CI 1.02–1.11) than to continue exercising below clinical guidelines over the year. Those with lower global function were less likely to experience positive changes in PA over the following year, nor to meet clinical guidelines at both baseline and 12 months than to continue exercising below clinical guidelines over the year, but this association did not withstand multiple testing. No differential effect of baseline function on change in PA levels was observed in the IMPaCT therapy group.

No significant associations between baseline depression severity and change in PA were found. Baseline mental health symptoms were not associated with changes in sedentary behaviour.

## Discussion

This study set out to describe the dietary intake, PA and sedentary behaviour patterns of a sample of patients with established psychosis. Moreover, we sought to explore the relationship between negative symptoms, depression severity and global function with these lifestyle factors through cross-sectional associations, and to explore associations between mental health symptomology and these lifestyle factors at future time points, and changes in these lifestyle factors over 12 months. This study found that a majority of the sample had poor dietary quality, low in fruit and vegetables and high in discretionary foods. Moreover, only 29.3% met PA guidelines of ⩾150 min of activity per week while 72.2% spent ⩾6 h per day sitting.

Those receiving IMPaCT therapy with more negative symptoms at baseline were less likely to meet PA recommendations at 12 months, at which time point, there was a significant cross-sectional relationship between PA and negative symptoms. Those with more negative symptoms receiving IMPaCT therapy also had fewer positive changes in PA from baseline to 12-month follow-up than those with fewer negative symptoms at baseline. These associations were not found in the TAU group.

Those with lower global function at baseline had higher levels of sedentary behaviour. Those with lower baseline global function receiving TAU were less likely to meet PA recommendations at 12-months.

Associations concerning depression severity and dietary intake did not withstand multiple testing. Moreover, the 12-month analyses were more likely to fail to withstand multiple testing due to the smaller sample size.

Poor dietary intake and limited activity in psychosis has been postulated as being linked to the lethargy, avolition and disturbances in planning and organisational capability associated with psychosis, which may contribute to reduced motivation and ability to plan and prepare meals (Albaugh, Singareddy, Mauger, & Lynch, 2011; Elman, Borsook, & Lukas, 2006; Firth et al., 2018a; Mangurian, Sreshta, & Seligman, 2013). This hypothesis is strengthened by the associations we found between negative symptoms and global function with activity, although findings between negative symptoms and global function with diet did not withstand correction for multiple testing which may be a feature of the complexity of this measure. Dietary choices may also be influenced by financial constraints and the side effects of psychotropic medication, particularly second-generation antipsychotic medication (including excessive hunger and cravings for high-calorie food) (Teasdale et al., 2019). The majority of this group (94%) was receiving psychotropic medication (Gardner-Sood et al., 2015).

Poor dietary intake, low levels of PA and high levels of sedentary behaviour are lifestyle factors that can cause or aggravate cardiometabolic diseases, contribute to years lived with disability and premature mortality (Gakidou et al., 2017). We have shown high levels of these lifestyle risk factors in a large sample of patients with established psychosis. These findings have important clinical implications. Psychiatric practitioners should make diet and exercise part of the overall treatment regime to offset the almost inevitable poorer physical health outcomes associated with having a severe and enduring psychosis. These results highlight the need for the development and implementation of effective and practical multidisciplinary lifestyle and exercise interventions to target eating habits, PA and sedentary behaviour in established psychosis in the hope of reversing cardiometabolic risk.

IMPaCT therapy, which used MI and CBT techniques, did not alter lifestyle risk factors in the overall analysis (Gaughran et al., 2017). In this study, when examining for the effect of baseline mental status, those with lower baseline global function were less likely to be in the 150 min+ category for PA at 12 months and were less likely to show change in activity levels when receiving TAU, but this impediment did not apply in the group receiving IMPACT therapy, suggesting that lower function at baseline did not impede any effectiveness of IMPaCT therapy. Negative symptoms however, appeared to pose more of a challenge. In IMPaCT, there was an association between more negative symptoms at baseline and persistent sub-threshold PA levels, with those who increased their exercise to target having fewer negative symptoms at the outset, although this association was not found in the TAU group. There was little differential effect of baseline depressive symptoms.

These varying effects of baseline mental health status on change in lifestyle factors in response to an intervention highlight the diversity of needs in people with psychosis and the consequent challenges of evaluating and comparing lifestyle interventions aimed towards this group. It also raises the possibility that those with negative symptoms in particular may require more tailored approaches to effect lifestyle change.

In other settings, multiple multidisciplinary lifestyle interventions to improve diet and PA have been trialled including cooking programmes (Lovell et al., 2014), dietetic consultations (Teasdale et al., 2014, 2016), exercise interventions (Romain et al., 2018; Rosenbaum et al., 2016) and peer-support platforms, smartphone apps and fitness trackers (Aschbrenner, Naslund, Shevenell, Kinney, & Bartels, 2016; Macias et al., 2015; Muralidharan et al., 2018). Future research will be needed to determine which forms of intervention, modes of delivery and levels of support are needed for optimal adherence and effectiveness in a given person with psychosis to combat poor lifestyle factors and cardiometabolic disease risk (Firth et al., 2019).

Our data suggested that those with more severe negative symptoms partake in low levels of PA. These results may suggest that future trials of interventions aimed at improving the lifestyle of those with established psychosis may need to be tailored to the symptom profile of individual patients, paying particular attention to negative symptoms. Additionally, future research evaluating treatments for negative symptoms should evaluate the effect on lifestyle metrics alongside. It has been proposed that PA should be assessed as a vital sign in every contact with a health professional (Sallis, 2011). Moreover, smartphones, wearable devices (Firth et al., 2018b; Naslund & Aschbrenner, 2019) and prospective record keeping could provide accurate methods of lifestyle assessment, although there is the need for exploration for the validity of these methods in people with SMI and the need to look for innovative methods that explore eating behaviours as well as dietary intake (Teasdale et al., 2019).

Finally, bidirectional relationships have been postulated between mental health symptomatology and both PA and nutrition (Da Silva et al., 2012; Raudsepp, 2016). Exercise can stimulate the serotonin system, dopaminergic system, hypothalamic–pituitary–adrenal (HPA) axis and increase brain-derived neurotrophic factor (BDNF) expression and potentially influence hippocampal volume and connectivity (Adlard, Perreau, & Cotman, 2005; Droste et al., 2003; Kandola, Ashdown-Franks, Hendrikse, Sabiston, & Stubbs, 2019; Weicker & Strüder, 2001). Moreover, the high antioxidant content in fruit and vegetables may reduce oxidative stress and decrease the risk of depression (Rahe, Unrath, & Berger, 2014). High intake of fruit and vegetables, and low intake of saturated fat, has also been postulated to affect mental health via modulation of inflammatory pathways, epigenetics, the HPA axis, BDNF expression, tryptophan–kynurenine metabolism and gut microbiota (Marx et al., 2021), although further research is needed into this relationship in people with psychosis (Van Der Pols, 2018).

### Limitations

Limitations of this study include the self-report nature of dietary and PA measures. Both the DINE and IPAQ required participants to detail their behaviour patterns from the previous week, thus relying on memory and cognitive function which may be impaired in established psychosis (Bora, Yücel, & Pantelis, 2010; Zanello, Curtis, Badan Bâ, & Merlo, 2009). Self-report may also be subject to social desirability biases and one's own interpretation (Podsakoff & Organ, 1986). To overcome these potential barriers, measures were completed in the presence of a mental health researcher and additional clarification was given regarding serving sizes and examples of foods when completing the DINE. Nonetheless, in future research digital technologies and food and exercise diaries could supplement questionnaire findings. Second, only fruit and vegetables were included in the correlational analysis for dietary factors due to limitations with the DINE in accurately quantifying food sources and mineral/nutrient intake. Furthermore, although other food groups have been associated with mental health, the WHO has set international recommendations for fruit and vegetable intake whereas recommendations for other food groups lack consensus. Future analyses should address overall dietary patterns and overall diet quality (Firth et al., 2020). Additionally, assessment of fruit and vegetables intake was based on weekly consumption at low amounts (i.e. <5 servings a week; ⩾5 weekly) due to the categorical nature of the DINE questionnaire. Future research should analyse fruit and vegetables intake in line with daily intake recommendations. Third, correlational analyses may have been mediated by the impact of cardiometabolic risk. In an analysis of IMPaCT baseline data (Gardner-Sood et al., 2015), hypertension and obesity were independently associated with GAF score and obesity was associated with depressive symptomatology. That said, no associations between cardiometabolic risk factors and PANSS score were found, and the baseline distribution of cardiometabolic risk factors was similar between treatment groups. Fourth, depression and negative symptoms include low energy and/or reduced activity, thus people with less PA or more sedentary behaviour may be more likely to meet these criteria. For example, the MADRS scale includes the item lassitude or difficulty initiating everyday tasks, and the PANSS negative scale includes the item passive/apathetic social withdrawal. Correlations between PA, negative symptoms and depression severity must be interpreted with caution.

Fifth, 22% of patients in IMPaCT RCT did not have 12-month data (Gaughran et al., 2013, 2017), raising the possibility of selective retention. However, there were similar mean scores for clinical characteristics, physical health measures and dietary intake between the total sample at baseline and those with 12-month follow-up data (Gaughran et al., 2017). Finally, the IMPaCT sample was limited to individuals with established psychosis, who had experienced multiple psychotic episodes, and who were receiving secondary care; they therefore required greater clinical support than those receiving primary care in London. The findings, therefore, may not be generalisable to those with early psychosis or less severe psychotic symptoms (Gardner-Sood et al., 2015). That said, a recent prospective study with 293 UK adults with FEP found that 57% consumed a carbonated drink daily, 66.5% added salt to food during cooking and 78.5% consumed take-away meals, three-quarters of whom did so more than once weekly. Additionally, 77% did not meet PA guidelines of 150 min per week and 57.3% sat for more than 6 h per day (Gaughran et al., 2019). Thus, it appears that similar eating behaviours and patterns of PA and sedentary behaviour are found during early psychosis, although further research is needed.

## Conclusion

This research has highlighted high levels of lifestyle risk factors, namely low fruit and vegetable consumption and PA and high levels of sedentary behaviour, in a sample of individuals with established psychosis, and has shown that various mental health symptoms may be associated with these poor lifestyle factors and negative symptoms in particular may reduce patient's responses to lifestyle interventions. Future research should evaluate the effect of treating mental health symptoms on lifestyle metrics. It also highlights a potential need for further research on how to adapt lifestyle interventions to baseline mental status and how to take such factors into account when planning evaluation strategies.

## Supplementary material

For supplementary material accompanying this paper visit https://doi.org/10.1017/S0033291721003147. click here to view supplementary material
