# Supplementary material for: Dietary intake, physical activity and sedentary behaviour patterns in a sample with established psychosis and associations with mental health symptomatology
Source: Psychol Med. 2021 Aug 23;53(4):1565–75. doi: 10.1017/S0033291721003147 (PMC10009388; doi:10.1017/S0033291721003147)
Supplement: Supplementary file 1 [file S0033291721003147sup001.docx]

**Appendix One- Modified Dietary Instrument for Nutrition Education (DINE)**

| 1. About how many pieces or slices per day do you eat of the following types of bread, rolls, or chapattis? (Choose one answer on each line) | | | | | |
| --- | --- | --- | --- | --- | --- |
| Breads & Rolls | None | Less than 1 a day | 1 to 2 a day | 3 to 4 a day | 5 or more a day |
| White bread or rolls (hard dough bread, white chapattis or parathas, West Indian bread) |  |  |  |  |  |
| Brown or granary bread or rolls (brown chapattis or parathas) |  |  |  |  |  |
| Wholemeal bread or rolls (wholemeal chapattis or parathas) |  |  |  |  |  |

| 2. About how many servings per week do you eat of the following types of breakfast cereal or porridge? (Choose one answer on each line) | | | | | |
| --- | --- | --- | --- | --- | --- |
| Breakfast cereals | None | Less than 1 a week | 1 to 2 a week | 3 to 5 a week | 6 or more a week |
| Sugared type: Frosties, Coco Pops, Ricicles Sugar Puffs; Rice or Corn type: Corn Flakes, Rice Krispies, Special K |  |  |  |  |  |
| Porridge or Ready Brek, Coco, Gari,  Wheat type: Shredded Wheat, Start, Weetabix, Fruit ‘n Fibre, Puffed Wheat Muesli type: Alpen, Jordan’s |  |  |  |  |  |
| Bran type: All-Bran, Bran Flakes, Country Bran |  |  |  |  |  |

| 3. About how many servings per week do you eat of the following foods? (Choose one answer on each line) | | | | | | | |
| --- | --- | --- | --- | --- | --- | --- | --- |
| Starchy Foods, Vegetables and Fruits | None | Less than 1 a week | 1 to 2 a week | 3 to 5 a week | 6 to 7 a week | 8 to 11 a week | 12 or more a week |
| Pasta or rice (include plain boiled rice, rice and peas, pillau, biryani, Jollof rice) |  |  |  |  |  |  |  |
| Potatoes – yams, dasheen, green banana, cassava, plaintains, breadfruit sweet potatoes, co-co, farina, dumplings, cornmeal, Fu Fu, Gari, or taro/eddo (excluding chips) |  |  |  |  |  |  |  |
| Green peas (3Tbs) |  |  |  |  |  |  |  |
| Beans (baked, tinned, or dried), lentils (dhal), pulses (150 – 200g = ½ tin) |  |  |  |  |  |  |  |
| Other vegetables (any type cooked or raw as in salads: carrots, cucumber, lettuce, tomatoes, celery, ackee, spinach, callaloo, okra, christophene) |  |  |  |  |  |  |  |
| Fruit (including fresh, frozen, or canned fruit: apples bananas, pears, peaches, mango, passion fruit, paw paw etc) |  |  |  |  |  |  |  |

Portion size guide for fruit and vegetables:

| Food Item | Portion Size |
| --- | --- |
| Vegetables (fresh, frozen or canned) | 3 tablespoons |
| Salad | 1 cereal bowlful |
| Vegetables in composites, such as vegetable chilli | 3 tablespoons |
| Very large fruit, such as a melon | 1 average slice |
| Large fruit, such as a grapefruit | ½ a fruit |
| Medium fruit, such as apples / oranges / bananas | 1 fruit |
| Small fruit, such as a plum, kiwi, apricot | 2 fruits |
| Very small fruit, such as blueberries/strawberries | 1 average handful |
| Dried fruit | 1 tablespoon |
| Frozen fruit/canned fruit | 3 tablespoons |
| Fruit in composites, such as stewed fruit in apple pie | 3 tablespoons |
| Fruit Juice | 1 small glass (150ml) |

| 4. About how many servings per week do you eat of the following foods? (Choose one answer on each line) | | | | | |
| --- | --- | --- | --- | --- | --- |
|  | None | Less than 1 a week | 1 to 2 a week | 3 to 5 a week | 6 or more a week |
| Cheese (any except cottage) |  |  |  |  |  |
| Beefburgers or sausages |  |  |  |  |  |
| Beef, pork, or lamb or goat (for vegetarians: nuts i.e. peanuts = groundnuts) |  |  |  |  |  |
| Bacon, meat pie, processed meat |  |  |  |  |  |
| Chicken or turkey (including processed chicken or turkey |  |  |  |  |  |
| Fish (NOT fried fish) |  |  |  |  |  |
| ANY fried food: fried fish i.e. salt fish, chips, cooked breakfast, samosas, West Indian soup or stew, fritters, fried potatoes, fried plantain, fried rice, puris, bhajis, plantain crisps |  |  |  |  |  |
| Cakes, pies, puddings, pastries or Indian sweets (e.g. ladoos, kheer). |  |  |  |  |  |
| Biscuits, chocolate, or crisps (including savoury biscuits such as cream crackers, Bombay mix, sev, chanachur). |  |  |  |  |  |

| 5. About how much of the following types of milk do you yourself use per day, for example in cereal, tea, or coffee? (Choose one answer on each line) | | | | | |
| --- | --- | --- | --- | --- | --- |
| Milk | None | Less than a quarter pint | About a quarter pint | About half a pint | 1 pint or more |
| Full cream (blue top) or  Channel Islands, Evaporated milk (regular and light versions), condensed milk |  |  |  |  |  |
| Semi-skimmed (green top) including dried semi-skimmed milk powder |  |  |  |  |  |
| Skimmed (red top) including dried skimmed, Boots dried powder, Co-Op powder |  |  |  |  |  |

| 6. About how many rounded teaspoons per day do you usually use of the following types of spreads, for example on bread, sandwiches, toast, potatoes, or vegetables? (Choose one answer on each line) | | | | | | | | |
| --- | --- | --- | --- | --- | --- | --- | --- | --- |
| Spreads | None | 1 | 2 | 3 | 4 | 5 | 6 | 7 or more |
| Regular margarine or butter or ghee or Regular (not reduced in fat) fat spread such as sunflower or olive spread, Flora, Vitalite, Clover, Olivio, Stork, Utterly Butterly |  |  |  |  |  |  |  |  |
| Low fat spread such as  Flora Light, St. Ivel Gold, Half-fat butter, Olivite, Flora Pro-activ, Light spread |  |  |  |  |  |  |  |  |

| 7. What type of fat do you usually use for the following purposes?  (Choose one answer on each line) | | | | | |
| --- | --- | --- | --- | --- | --- |
|  | Butter, lard, or dripping or coconut oil/cream  or palm kernel oil  or ghee | Solid cooking fat (White Flora, Cookeen )  Half-fat butter  Hard margarine (Stork) | Soft margarine  (sunflower, soya)  Regular (not reducedin fat) fat spread (olive, Flora Buttery, Olivio) | Vegetable oil or Low fat spread (Flora Light, Olivite, St. Ivel Gold) | No fat used |
| On bread and vegetables |  |  |  |  |  |
| For frying |  |  |  |  |  |
| For baking or cooking |  |  |  |  |  |

ADDITIONAL QUESTIONS

| 8. How often do you eat fresh fruits or vegetables? | | | | | | |
| --- | --- | --- | --- | --- | --- | --- |
| Seldom or never | Less than once a month | 1 – 3 times a month | 1- 2 times a week | 3 – 4 times a week | 5 – 6 times a week | 7 or more times a week |
|  |  |  |  |  |  |  |

| 9. Has salt been generally added to your food during cooking? | | |
| --- | --- | --- |
| Yes (include sea salt) | No, do not use salt in cooking | Use ‘Lo Salt’ or salt alternative |
|  |  |  |

| 10. At the table do you: | | | |
| --- | --- | --- | --- |
| Generally add salt to your food without tasting it first? | Taste the food, but then generally add salt? | Taste the food, but only occasionally add salt? | Rarely or never add salt at the table |
|  |  |  |  |

| 11. About how much per week do you drink of the following soft drinks? (Choose one answer on each line) | | | | | |
| --- | --- | --- | --- | --- | --- |
| Soft drinks | None | Less than 1 a week | 1 to 2 a week | 3 to 5 a week | 6 or more a week |
| Fizzy drinks (Coca-cola, Pepsi, Sprite) 1 litre bottles |  |  |  |  |  |
| Diet Fizzy drinks (Diet coke, diet Pepsi, diet Sprite) 1 litre bottles |  |  |  |  |  |
| Fruit Juices (orange juice, apple, pineapple etc) 500 ml |  |  |  |  |  |
| Nurishment (cans – 420g) |  |  |  |  |  |
| Supermalt (bottles – 330ml) |  |  |  |  |  |

| 12. How many cups of the following beverages do you drink per day? | | | | | |
| --- | --- | --- | --- | --- | --- |
| Beverages | None | 1 cup a day | 2 to 3 cups a day | 4 to 5 a day | 6 or more cups a day |
| Tea |  |  |  |  |  |
| Coffee |  |  |  |  |  |
| Hot chocolate |  |  |  |  |  |
| Herbal teas (fruit tea, camomile, peppermint etc) |  |  |  |  |  |

| 13. How many spoons of sugar do you add to each cup of hot drink? | | | | | |
| --- | --- | --- | --- | --- | --- |
| Sugar | No sugar | 1 tsp | 2 tsp | 3 tsp | 4 or more tsps |
| Tea |  |  |  |  |  |
| Coffee |  |  |  |  |  |
| Hot chocolate |  |  |  |  |  |
| Herbal teas (fruit tea, camomile, peppermint etc) |  |  |  |  |  |

| 14. How often do you eat takeaway meals? (e.g. fried chicken, fish & chips, Indian, Chinese, pizza, burgers) | | | | | | |
| --- | --- | --- | --- | --- | --- | --- |
| Seldom or never | 1- 2 times a week | 3 – 4 times a week | 5 – 6 times a week | 7 or more times a week | 1 – 3 times a month | Less than once a month |
|  |  |  |  |  |  |  |
